# Contrast Medium Induced Nephropathy after Endovascular Stent Graft Placement: An Examination of Its Prevalence and Risk Factors

**DOI:** 10.1155/2016/5950986

**Published:** 2016-03-16

**Authors:** Yohei Kawatani, Yoshitsugu Nakamura, Yoshihiko Mochida, Naoya Yamauchi, Yujiro Hayashi, Tetsuyoshi Taneichi, Yujiro Ito, Hirotsugu Kurobe, Yuji Suda, Takaki Hori

**Affiliations:** ^1^Department of Cardiovascular Surgery, Chiba-Nishi General Hospital, 107-1 Kanegasaku Matsudo-Shi, Chiba-Ken 2702251, Japan; ^2^Department of Clinical Engineer, Chiba-Nishi General Hospital, 107-1 Kanegasaku Matsudo-Shi, Chiba-Ken 2702251, Japan

## Abstract

Endovascular stent graft placement has become a major treatment for thoracic and abdominal aneurysms. While endovascular therapy is less invasive than open surgery, it involves the use of a contrast medium. Contrast media can cause renal impairment, a condition termed as contrast-induced nephropathy (CIN). This study sought to evaluate the incidence and risk factors of CIN following endovascular stent graft placement for aortic aneurysm repair. The study included 167 consecutive patients who underwent endovascular stent graft placement in our hospital from October 2013 to June 2014. CIN was diagnosed using the European Society of Urogenital Radiology criteria. Patients with and without CIN were compared. Chi-squared tests, *t*-tests, and multivariate logistic regression analyses were performed. Thirteen patients (7.8%) developed CIN. Left ventricular dysfunction and intraoperative blood transfusion were significantly more frequent in the CIN group (*P* = 0.017 and *P* = 0.032, resp.). Multivariate analysis showed that left ventricular dysfunction had the strongest influence on CIN development (odds ratio 9.34, *P* = 0.018, and 95% CI = 1.46–59.7). Patients with CIN also experienced longer ICU and hospital stays. Measures to improve renal perfusion flow should be considered for patients with left ventricular dysfunction who are undergoing endovascular stent graft placement.

## 1. Introduction

Compared with open repair, endovascular stent graft placement is a minimally invasive procedure with low rates of perioperative mortality and complications, including low rates of renal dysfunction [1, 2]. However, one invasive element of endovascular stent graft placement that is not present in open repair is the intraoperative use of contrast media. Contrast media are known to damage the kidneys, and the resulting nephropathy is a complication of endovascular stent graft placement. Renal damage that occurs due to the use of a contrast medium is referred to as contrast-induced nephropathy (CIN).

CIN is the third most common cause of renal failure that occurs during hospitalization [3]. In various fields, reports state that CIN-related renal dysfunction is associated with worse outcomes, including high mortality rates and prolonged hospitalization stays [4, 5]. Although there are many reports concerning CIN, the reported prevalence rates differ due to differences in the diagnostic criteria used [6]. There are few reports with clear diagnostic criteria concerning CIN associated with endovascular stent graft placement. Here, we examine the prevalence of CIN due to endovascular stent graft placement in our hospital and determine the risk factors for CIN, based on the European Society of Urogenital Radiology diagnostic criteria [7].

## 2. Patients and Methods

### 2.1. Clinical Data Collection

The present study included 184 patients who underwent endovascular stent graft placement in our hospital from October 2013 to June 2014. Of these, we excluded 12 patients who received operative treatment for aortic aneurysm rupture, three patients in whom acute aortic dissection was performed, one patient who received operative treatment including thoracotomy, and one patient with chronic renal failure who had received preoperative hemodialysis.

All clinical data were collected from the patients' medical records.

We utilized the CIN diagnostic criteria of the European Society of Urogenital Radiology, as follows: “a condition in which a decrease in renal function occurs within three days of the intravascular administration of a CM in the absence of an alternative etiology. An increase in serum creatinine by more than 25% or 44 *μ*mol/L (0.5 mg/dL) indicates CIN” [7].

Data related to the patients' demographics and preoperative status were collected, including age, gender, and past medical history, such as hypertension (patients receiving oral therapy or untreated cases), diabetes (patients receiving insulin), and impaired left ventricular function (30% or lower ejection fraction). The serum creatinine value obtained from the most recent preoperative blood test before the day of operative treatment was used as the preoperative creatinine level, and, postoperatively, the highest value obtained during the first to third postoperative days was defined as the postoperative creatinine level. We measured the serum creatinine levels and predicted the glomerular filtration rates (eGFR) using the calculation method recommended by the Japanese Society of Nephrology [8].

We examined the following factors related to operative treatment: type of procedure (thoracic or abdominal endovascular stent graft placement), operative time, quantity of contrast medium used, and intraoperative red blood cell transfusion. Treatment outcomes were in-hospital death, renal replacement therapy, length of ICU stay, and length of postoperative hospitalization.

All patients undergoing surgery at our facility were given an explanation of the significance of publishing their clinical data at academic meetings or in the scientific literature, and all patients in this study provided informed consent to participate in studies conducted in our facility.

### 2.2. Perioperative and Intraoperative Management

In our hospital, patients whose preoperative blood test revealed a serum creatinine level greater than 1.3 mg/dL were hospitalized three days preoperatively and received Ringer's lactate solution (60 mL/h) intravenously. The initiation of postoperative food intake was accompanied by the termination of reinfusion fluid, but in cases in which serum creatinine levels remained elevated, hydration was continued until improvement was observed.

Inhalational anesthetic agents were used to induce general anesthesia in all patients. Fentanyl and remifentanil were used as analgesics. Eslax (rocuronium bromide) was used as a muscle relaxant. Perioperative fluid management, including fluid control and hemodynamic monitoring during the operation, was performed by an anesthesiologist.

Iopamidol was used as an intraoperative contrast medium.

The access route for stent graft insertion was the femoral artery via an inguinal incision; a sheath was placed in the artery and the stent graft was inserted.

All patients awoke in the operating room and were subsequently transferred to the ICU.

### 2.3. Statistical Analysis

Continuous variables are presented as the mean ± standard deviation, and categorical variables are presented as the total (percentage [%]). Continuous variables such as age, surgical duration, amount of contrast medium used, and length of stay in the ICU and hospital were analyzed using Student's *t*-tests. Chi-squared tests were used to compare categorical variables such as gender, medical history, presence or absence of intraoperative packed red blood cell transfusion, and presence or absence of transcatheter arterial embolization of the internal iliac artery. Differences were considered statistically significant when the *P* value < 0.05.

We performed a multiple logistic regression analysis that included all items with a *P* value < 0.05 in the univariate analyses to examine the factors with the greatest influence on the onset of CIN.

All statistical analyses were performed on a personal computer using the statistical software package SPSS for Mac (Version 22; SPSS Inc., Chicago, IL, USA).

## 3. Results

After excluding patients who met the exclusion criteria, this study included 167 patients. CIN was observed in 13 patients (7.8%). No cases of acute renal failure that required postoperative renal replacement therapy were observed. No significant differences in age, gender, history of hypertension, surgical procedure, preoperative serum creatinine level, or preoperative eGFR were observed between the CIN and non-CIN groups. Three patients (23%) in the CIN onset group had impaired left ventricular function, compared with five patients (3.2%) in the non-CIN group; thus, impaired left ventricular function was significantly more prevalent in the CIN onset group (*P* = 0.017; Table 1). Changes between pre- and postoperative creatinine and eGFR were the greatest in patients in whom CIN developed. Intraoperative blood transfusion was performed in three patients (23%) in the CIN onset group and seven patients (4.5%) in the non-CIN group; this was significantly more frequent in the CIN onset group (*P* = 0.032). No significant differences were noted for operative time or quantity of contrast medium used (Table 2). The difference in the mean length of ICU stay was 1.11 days, and the difference in the mean length of hospitalization was eight days; the group in which CIN developed experienced significantly longer ICU and hospital stays (Table 3).

The results of a multiple logistic regression analysis revealed that impaired left ventricular function was the risk factor with the greatest impact on the development of CIN (Table 4).

## 4. Discussion

Although CIN has been examined in many studies, several mechanistic aspects of the disease remain unclear. Structural changes caused by the contrast medium include vacuolar degeneration of the proximal tubule cells, DNA fragmentation, and necrosis in the ascending limb of the loop of Henle [4]. There are also reports concerning its relationship with interstitial inflammatory reactions [5]. The reduction in the renal perfusion flow of the contrast medium due to the effects of CIN is also thought to be a cause. It is thought that when the kidney is exposed to a contrast medium, vasoconstriction-related hypoxia of the renal medulla occurs, causing nephropathy associated with the cellular metabolism of the renal tubules [6]. Reports have also indicated that CIN plays a role in decreased cardiac output and vasopressin secretion [8].

Li et al. examined CIN associated with percutaneous coronary intervention (PCI) used to treat heart failure and found that a past history of heart failure is a risk factor for CIN [9]. Additionally, the results of a study by da Silva Selistre et al. analyzing patients who received contrast-enhanced computed tomography (CT) similarly showed that a past history of heart failure is a risk factor for CIN [10]. Echocardiography was used to assess cardiac function in the present study. Impaired left ventricular function revealed by preoperative echocardiography was found to be a risk factor. A multivariate analysis showed that the odds ratio of the most influential risk factor was 9.34.

When decreased renal perfusion flow caused by a contrast medium occurs, patients with impaired left ventricular function have poorer perfusion flow compared to those with normal ventricular function. This may be why it is a risk factor for CIN onset.

These findings suggest that the intraoperative and postoperative use of catecholamines to increase cardiac output and improve the patient's hemodynamics and renal perfusion flow could prevent and treat CIN in patients with impaired left ventricular function. This should be explored in future studies.

Intraoperative blood transfusion was a risk factor for CIN. There are no reports concerning CIN associated with procedures in which blood transfusions were necessary. In the present study, only 1.80% of the total number of patients needed a blood transfusion; thus blood transfusions were generally not performed during endovascular stent graft placement. It appears that, in such procedures, intraoperative hemodynamic instability requiring blood transfusions led to a decrease in renal perfusion flow. No intraoperative aneurysm ruptures or conversions to open repair occurred during the study procedures. In our experience, the most common sites of hemorrhage during endovascular stent graft placement in patients who do not have a ruptured aortic aneurysm are the sheath placement site and the sheath retraction site. From the viewpoint of CIN prevention, it is necessary to thoroughly manage the sheath placement site in order to limit the amount of intraoperative hemorrhaging, as well as to retract the sheath with extreme care during the procedure.

It is thought that intravenous hydration provides effective prophylaxis against CIN [11]. A study by Luo et al. of ST-segment elevation myocardial infarction (STEMI) patients who were randomly assigned to receive PCI reported that hydration following treatment lowered the incidence of CIN and improved short-term prognosis [12]. It is believed that, by increasing renal blood flow and urinary flow through hydration, it is possible to reduce the renal exposure to contrast media [11]. In our hospital, Ringer's lactate solution is used for preoperative intravenous hydration, but solutions such as 0.45% saline, 0.9% saline, and sodium bicarbonate have also been proposed. A previous randomized, controlled trial comparing the use of saline and sodium bicarbonate reported that sodium bicarbonate provides superior preventive effects [13]. Moreover, preoperative oral fluid intake inhibits decreases in eGFR [14]. Further examination and modification of infusion contents and hydration methods are needed.

A high preoperative creatinine level has been reported to be a risk factor for CIN [15]. However, in the case of endovascular aortic/aneurysm repair, one study reported that there was no relationship between serum creatinine level and the onset of CIN [16]. In the present study, a high creatinine level and low eGFR were not significant risk factors. Patients with a creatinine level of 1.3 or higher during the preoperative assessment in our hospital were assumed to be at a high risk for CIN; thus we performed intravenous hydration in such patients. Preoperative reinfusion fluid is effective in preventing CIN; thus this hospital procedure may have biased the study results in this regard. Furthermore, a limitation of the present study was that the total number of patients was small. Thus, further examination is needed.

It has been reported that the ratio of contrast volume divided by eGFR (CV/eGFR ratio) in PCI is a useful predictive factor of CIN [17]. A comparison of the CIN and non-CIN groups in the present study revealed that the CV/eGFR ratio was significantly higher in the CIN group. This suggests that a high CV/eGFR ratio may be a risk factor for CIN onset.

Additionally, because the CIN onset group tended to be elderly, in the present study we also examined the age × CV interaction. These values were also significantly higher in the CIN onset group; thus it appears that a high value for this parameter can also be a risk factor.

High-density contrast media are used during endovascular stent graft placement in our hospital. The use of a high-density contrast medium in animal experiments was reported to cause a higher level of renal impairment [18]. It is possible that, by changing to an isobaric or hypobaric contrast medium, the incidence of CIN can be further decreased.

CIN is associated with poor mortality and morbidity as well as an increased length of hospitalization [19]. In the present study, we examined short-term parameters, including the length of ICU stay and the length of postoperative hospital stay. The length of ICU stay and length of hospitalization of the CIN onset group were 3.77 ± 2.94 days and 19.8 ± 14.9 days, respectively: both were significantly longer than the group that did not develop CIN. The present study also concludes that CIN is associated with a poor prognosis.

It is possible that intravenous hydration prevents CIN: the results of the present study concerning impaired left ventricular function and intraoperative blood transfusion as risk factors are similar to the results of previous clinical studies from other fields and studies concerning the pathology of the condition. Factors leading to decreased renal perfusion flow are similarly considered to be risk factors for the development of CIN.

It is possible that, by obtaining the needed circulation serum and maintaining favorable intraoperative and perioperative hemodynamics, renal perfusion flow can be improved, leading to the prevention of CIN.

In the present study, our diagnoses were based on the definition of CIN put forth by the European Society of Urogenital Radiology. However, elements that are extremely difficult to evaluate, such as the effects of device manipulation and micro embolism proximal to the renal artery associated with the stent graft, are likely involved in the development of renal dysfunction occurring after stent graft placement. The inflammatory reaction is a well-known factor causing renal dysfunction, and inflammatory reactions also occur after placement of the stent graft [20]. Thus, further examination of the mechanism of CIN is needed.

## 5. Conclusion

Impaired left ventricular function and intraoperative blood transfusion were risk factors for CIN associated with endovascular stent graft placement. Impaired left ventricular function had the strongest influence on CIN risk. The CV/eGFR ratio and age × CV/eGFR ratio were significantly higher in the CIN onset group. The length of ICU stay and length of postoperative hospitalization tended to be longer in the CIN onset group. Preventive measures, such as catecholamine administration and intravenous hydration, should be explored for their use in patients with impaired left ventricular function who are undergoing endovascular stent graft placement.

## Figures and Tables

**Table 1:** Characteristics of patients who underwent endovascular stent graft placement.

	CIN	Non-CIN	Total	*P* value
Total number	13	154	167	
Age	77.7 ± 11.6	70.8 ± 9.55	71.3 ± 9.86	0.056
Males	12 [92.3%]	121 [73.8%]	133 [79.6%]	0.471
Hypertension	7 [53.8%]	84 [54.5%]	91 [54.5%]	1
Diabetes	0 [0.00%]	10 [6.50%]	10 [5.99%]	1
Left ventricular dysfunction	3 [23.1%]	5 [3.25%]	8 [4.79%]	0.017
Age 80 years or older	23 [15.0%]	7 [53.8%]	40 [24.0%]	0.02

Data are presented as the mean ± standard deviation or *N* [%]. CIN: contrast-induced nephropathy.

**Table 2:** Clinical parameters and outcomes.

Parameter	CIN	Non-CIN	Total	*P* value
Preoperative creatinine (mg/dL)	1.06 ± 0.364	0.91 ± 0.317	0.931 ± 0.332	0.191
Postoperative creatinine (mg/dL)	1.55 ± 0.600	0.883 ± 0.281	0.935 ± 0.362	<0.001
Changes in creatinine level (mg/dL)	0.490 ± 0.303	−0.036 ± 157	0.00451 ± 0.222	<0.001
Preoperative eGFR (mL/min/1.73 m^2^)	57.5 ± 19.9	65.1 ± 19.9	64.5 ± 19.4	0.211
Postoperative eGFR (mL/min/1.73 m^2^)	38.9 ± 14.4	67.5 ± 20.0	65.3 ± 21.0	<0.001
Changes in eGFR (mL/min/1.73 m^2^)	−18.7 ± 8.66	2.44 ± 9.43	0.797 ± 10.9	<0.001
Operative procedure (TEVAR)	7 [53.8%]	68 [44.2%]	75 [44.9%]	0.569
Intraoperative red blood cell transfusion	3 [23.1%]	7 [45.5%]	10 [59.9%]	0.032
Operative time (min)	125 ± 59.9	115 ± 60.0	118 ± 57.8	0.567
Successful endovascular treatment	13 [100%]	154 [100%]	167 [100%]	NS
CV/eGFR	2.23 ± 1.42	1.53 ± 0.777	1.589 ± 0.859	0.005
CV *∗* age	8560 ± 5129	6534 ± 3048	6691 ± 3280	0.032

Data are presented as the mean ± standard deviation or *N* [%]. CIN: contrast-induced nephropathy; eGFR: estimated glomerular filtration rate; TEVAR: thoracic endovascular aortic/aneurysm repair; NS: not significant; CV: contrast volume.

**Table 3:** Treatment outcomes.

Parameter	CIN	Non-CIN	Total	*P* value
In-hospital death	0	0	0	NS
RRT performed	0	0	0	NS
Length of ICU stay	3.77 ± 2.94	2.66 ± 1.66	2.77 ± 1.80	0.033
Length of hospitalization	19.8 ± 14.9	11.8 ± 1.66	12.4 ± 9.97	0.005

Data are presented as the mean ± standard deviation. CIN: contrast-induced nephropathy; NS: nonsignificant; RRT: renal replacement therapy; ICU: intensive care unit.

**Table 4:** Factors associated with the development of CIN.

	Odds ratio	*P* value	95% CI
Aged 80 years or older	7.80	0.026	1.28–47.4
Left ventricular dysfunction	9.34	0.018	1.46–59.7

CIN: contrast-induced nephropathy; CI: confidence interval. Note that left ventricular dysfunction was defined as an ejection fraction of 30% or lower.
